# Tissue-Engineered Solutions in Plastic and Reconstructive Surgery: Principles and Practice

**DOI:** 10.3389/fsurg.2017.00004

**Published:** 2017-02-23

**Authors:** Sarah Al-Himdani, Zita M. Jessop, Ayesha Al-Sabah, Emman Combellack, Amel Ibrahim, Shareen H. Doak, Andrew M. Hart, Charles W. Archer, Catherine A. Thornton, Iain S. Whitaker

**Affiliations:** ^1^Reconstructive Surgery and Regenerative Medicine Research Group (ReconRegen), Institute of Life Science, Swansea University Medical School, Swansea, UK; ^2^The Welsh Centre for Burns and Plastic Surgery, Morriston Hospital, Swansea, UK; ^3^Institute of Child Health, University College London, London, UK; ^4^In Vitro Toxicology Group, Institute of Life Science, Swansea University Medical School, Swansea, UK; ^5^Canniesburn Plastic Surgery Unit, Centre for Cell Engineering, University of Glasgow, Glasgow, UK; ^6^Cartilage Biology Research Group, Institute of Life Science, Swansea University Medical School, Swansea, UK; ^7^Human Immunology Group, Institute of Life Science, Swansea University Medical School, Swansea, UK

**Keywords:** tissue engineering, regenerative medicine, stem cells, translation, bioengineering, barriers to translation, translational research, plastic and reconstructive surgery

## Abstract

Recent advances in microsurgery, imaging, and transplantation have led to significant refinements in autologous reconstructive options; however, the morbidity of donor sites remains. This would be eliminated by successful clinical translation of tissue-engineered solutions into surgical practice. Plastic surgeons are uniquely placed to be intrinsically involved in the research and development of laboratory engineered tissues and their subsequent use. In this article, we present an overview of the field of tissue engineering, with the practicing plastic surgeon in mind. The Medical Research Council states that regenerative medicine and tissue engineering “holds the promise of revolutionizing patient care in the twenty-first century.” The UK government highlighted regenerative medicine as one of the key eight great technologies in their industrial strategy worthy of significant investment. The long-term aim of successful biomanufacture to repair composite defects depends on interdisciplinary collaboration between cell biologists, material scientists, engineers, and associated medical specialties; however currently, there is a current lack of coordination in the field as a whole. Barriers to translation are deep rooted at the basic science level, manifested by a lack of consensus on the ideal cell source, scaffold, molecular cues, and environment and manufacturing strategy. There is also insufficient understanding of the long-term safety and durability of tissue-engineered constructs. This review aims to highlight that individualized approaches to the field are not adequate, and research collaboratives will be essential to bring together differing areas of expertise to expedite future clinical translation. The use of tissue engineering in reconstructive surgery would result in a paradigm shift but it is important to maintain realistic expectations. It is generally accepted that it takes 20–30 years from the start of basic science research to clinical utility, demonstrated by contemporary treatments such as bone marrow transplantation. Although great advances have been made in the tissue engineering field, we highlight the barriers that need to be overcome before we see the routine use of tissue-engineered solutions.

## Introduction

Reconstructive plastic surgery aims to provide living tissue in order to restore both form and function following a wide range of congenital or acquired defects. Operations are complex, often transcending anatomic boundaries. Versatility resulting from surgery on a full range of tissues including skin, fat, nerve, muscle, bone, and cartilage promotes innovation, and with the recent advances in medical imaging (1), microsurgery (2), vascularized composite allotransplantation (3, 4), nanotechnology (5), cell biology, biomaterials (6), and 3D printing (7–10), treatment options for patients are wider than ever before. Even armed with new reconstructive options based on microsurgical principles and transplantation, surgeons have become increasingly cognizant that there is the real potential for a paradigm shift in reconstructive surgery in the medium term via tissue-engineered solutions. The implementation into practice could potentially eliminate the need for donor sites and their morbidity, reduce hospital stay and associated costs (11).

### Relevance of This Article

Contrary to public perception, the diverse workload of reconstructive plastic surgeons comprises a relatively small proportion of purely esthetic procedures (12). The majority of operations undertaken pertain to neoplasia and wound management, with a significant health economic impact (12). Over one million patients are treated per year in NHS England by Plastic Surgeons (13, 14), with evidence suggesting that this workload will continue to increase (15). If you extrapolate these figures worldwide, it is easy to see the clinical need is vast. As a group, reconstructive surgeons are facing more challenging composite defects than ever before coupled with Internet and media savvy patients with increasing expectations (16). Technological innovation in reconstructive surgery in the twentieth century offered the possibility for surgeons to operate on microvascular structures enabling free tissue transfers (2) and extremity replantations. Despite these developments in practice, we are still confronted with shortcomings relating to the availability of donor tissues. In order to overcome this, novel approaches have been investigated. Among these approaches, the most attractive concept is tissue engineering.

Tissue engineering is a modern, interdisciplinary field combining principles of engineering, physics, and the life sciences. It shares a common objective with plastic and reconstructive surgery; “to restore form and function” (17, 18). The long-term aim of tissue engineering is to biomanufacture autologous, vascularized, physiologically relevant solutions to repair and restore complex defects. Successful biomanufacture will depend on the correct blend of cell source, suitable scaffold and ideal microenvironment (19). Answers to these fundamental questions rely on interdisciplinary collaboration between cell biologists, material scientists, biotechnologists, and associated medical specialties (20). Upscaling and widespread use in health services will need close interaction with the cell therapy industry and associated manufacturers.

The surgical community worldwide is becoming increasingly aware of the research landscape. The American Society of Plastic surgeons have highlighted the role of tissue engineering in the future of plastic surgery (21), particularly the need for a focus on translation from bench research to clinical practice. In the United Kingdom, the House of Lords recognized the potential of regenerative medicine to impact on the health service and highlighted the current “lack of coordination” in the field as a whole (22, 23). Recommendations included the development of multidisciplinary working groups (basic scientists, clinicians, investors, manufacturing experts, and regulators), as well as governmental support to drive forward the agenda on regenerative medicine.

There is a need to develop NHS capacity with regional facilities licensed for Good Laboratory Practice (GLP) and current Good Manufacturing Practices (cGMP), which are engaged with a clinical specialty skilled in the manipulation of cells and the viable insertion of tissue-engineered constructs. Skilled in vascularization and in tissue viability/transfer, plastic surgeons already fulfill this role as an interface specialty delivering complex reconstructive techniques to a broad range of other specialties. The regional service structure of plastic surgery within the NHS would further support their capacity to align with regional cGMP facilities and deliver tissue-engineering solutions to a range of medical and surgical specialties.

### Objectives

#### Assess the Shortcomings Of

##### Traditional Reconstructive Options

Up till now, restoration of form and function has relied on the use of autologous (rarely allogeneic) tissue, alloplastic implants, or a combination of the two. Although effective, these options have disadvantages that merit highlighting (Table 1).

**Table 1:** **Advantages and shortcomings of reconstructive solutions for managing tissue defects**.

Reconstructive solution	Advantages	Disadvantages
Autologous	No immunological complications No ethical constraints Biologically compatible Minimal degradation Fewer legal restrictions No disease transmission Challenging harvesting cells in aged or diseased	Donor site morbidity Limited quantity of tissue available Two separate operative sites—greater risk and cost
Allogeneic	No donor site morbidity Donor cells may have higher viability Tissue always healthy Greater quantity of available tissue	Temporary (i.e., cadaveric skin used in extensive burns) Tissue typing is required Immunosuppression may be needed Risk of disease transmission Greater legal hurdles Ethical and psychological challenges
Synthetic	Maintain structural integrity Predictable and reproducible physical and mechanical properties Cost effective Avoids concerns over disease transmission	Extrusion Infection Cannot restore all of specialized tissue/organ functions Do not respond to biological cues/grow with patient May provoke an immune/inflammatory/fibrotic reaction Materials safety testing and manufacturing governance
Tissue engineered	Biocompatible Good biofunctionality Good retention of size and shape No donor site morbidity Unlimited expansion of cells/tissues No immunological concerns Mechanical stability	Long-term effect unknown Size often limited by vascularity Costly Tumorigenic potential Difficult to engineer “physiologically relevant/mature tissue”

##### Tissue-Engineered Solutions

**Figure 1:**
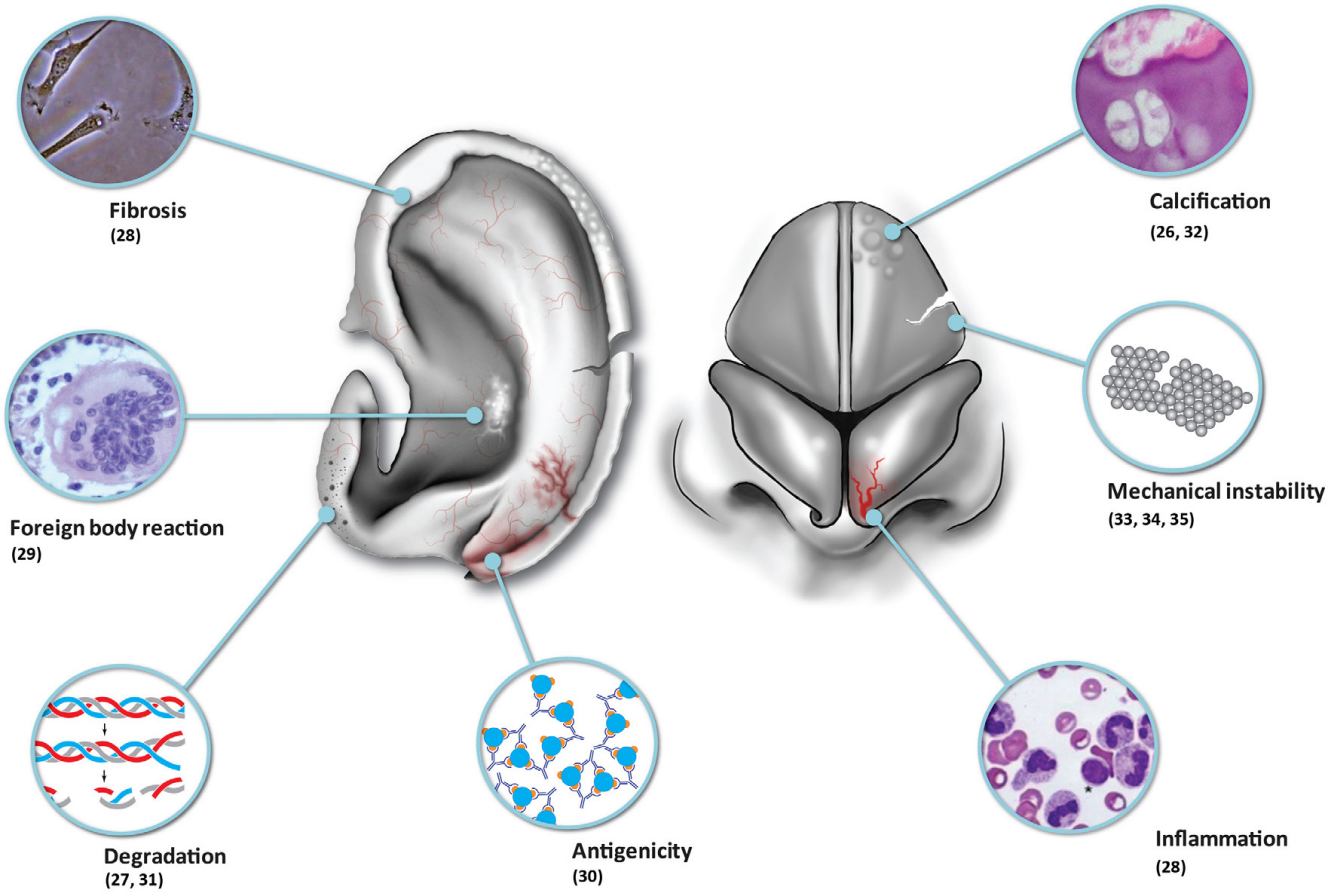
**Shortcomings of nasal and auricular cartilage tissue engineering**.

Although tissue-engineered solutions hold great promise, we must be realistic in that contemporary tissue-engineered constructs implanted into immune-competent animal models have been observed to undergo inflammation, fibrosis, foreign body reaction, and degradation (Table 1; Figure 1). One of the major problems remains vascularization of larger volume constructs (24), and an issue coming to the fore in modern literature is the potential for tumorigenesis (25).

#### Provide an Overview Of

##### Fundamental Principles of Tissue Engineering: Cell Source, Scaffold, Assembly Method, and Molecular and Mechanical Signaling

**Figure 2:**
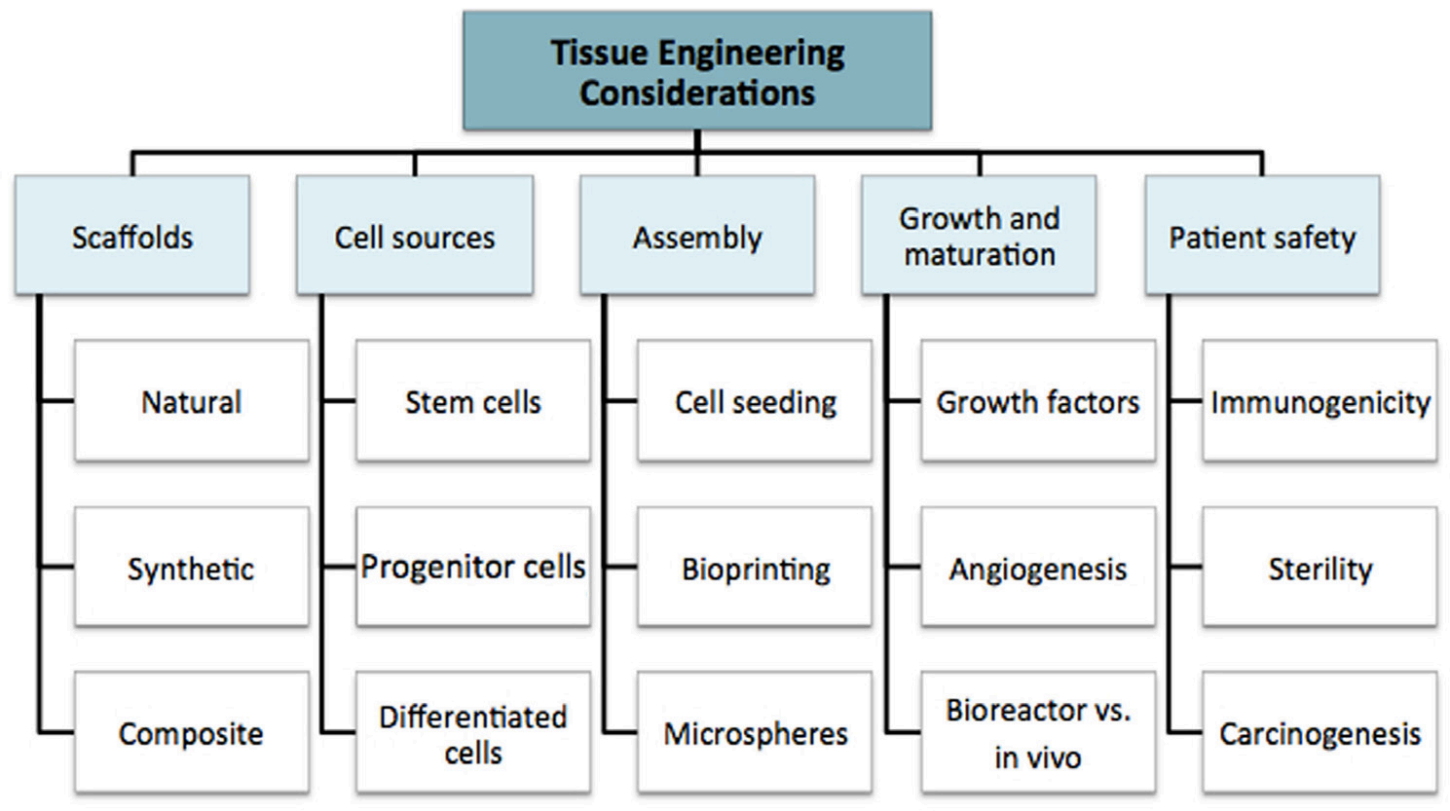
**Considerations in the field of tissue engineering**.

We discuss the multiple considerations for tissue engineering research in order to highlight the complexity of the field as a whole. This supports the argument for multidisciplinary coordination, which is required to take the field forward. The fundamental principles are summarized in Figure 2: cell source, scaffolds, assembly method, subsequent growth (molecular and mechanical signaling), and patient safety. These factors all contribute to the “environment” (19). In simple terms, the cell is required for synthesis of the new tissue matrix, while the scaffold, biomolecules, and the microenvironment provide trophic cues to guide proliferation and differentiation. Growth, induction, and maintenance of maturation are important for providing durability of the tissue-engineered construct.

##### Cell Sources

**Figure 3:**
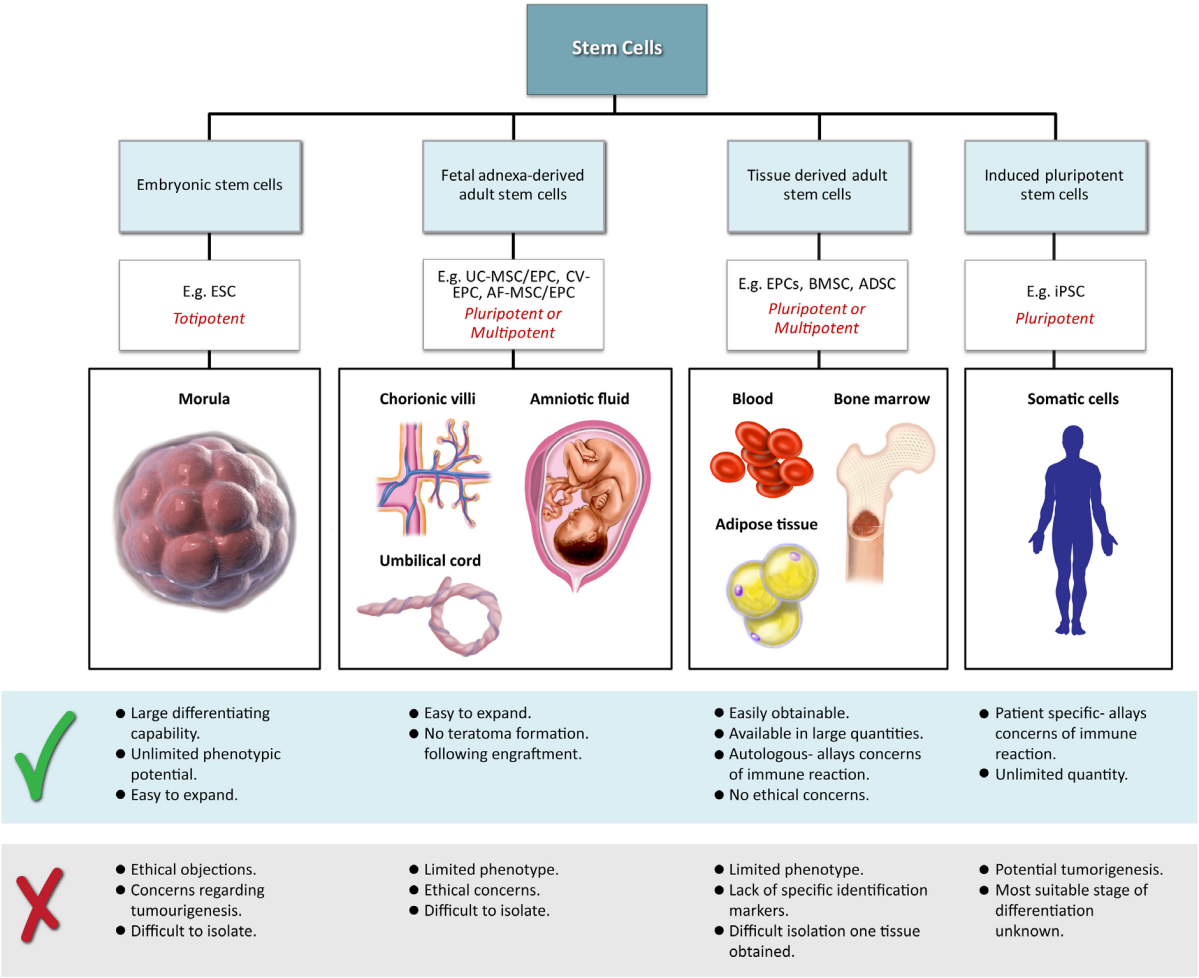
**Advantages and disadvantages of different cell sources utilized in tissue engineering**.

**Figure 4:**
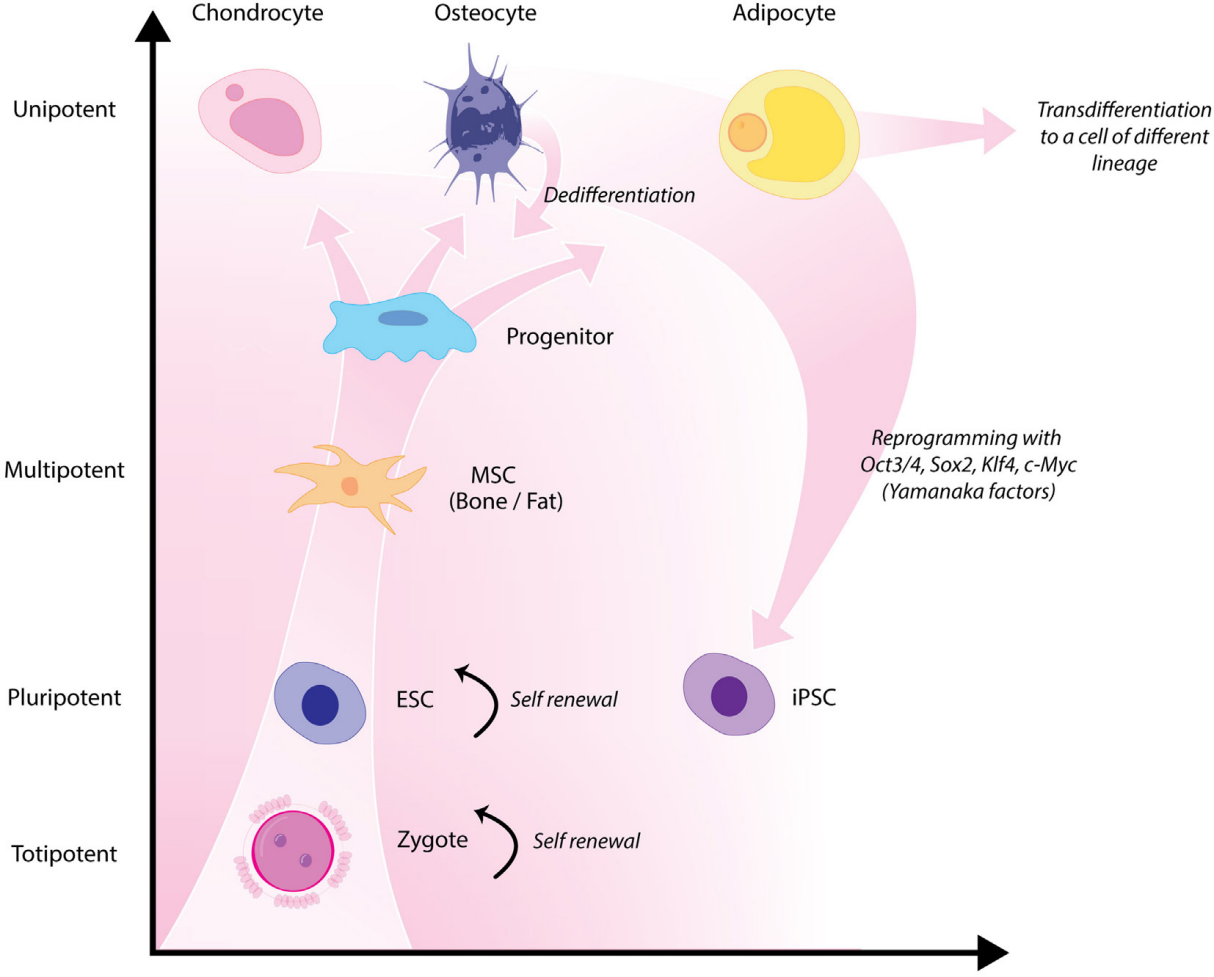
**Hierarchy of stem cells highlighting different degrees of potency**. Yamanaka factors are used to induce differentiated cells to become pluripotent.

Classical tissue-engineering approaches use *tissue-derived* cells (*not necessarily stem cells*) seeded onto scaffolds (36). These cells may be autologous, allogenic, or xenogenic cells; however, autologous cells are the preferred choice due to the lack of immunogenicity. Cells may be further classified based on differences in their differentiating capabilities (Figure 3). Adult somatic cells are fully differentiated, and therefore have restricted future differentiation potential and relatively poor growth, limiting their usefulness for tissue-engineering purposes (37). Progenitor cells are more differentiated than stem cells and are therefore referred to as multipotent rather than pluripotent (38). Stem cells are non-differentiated cells, able to proliferate through multiple generations and differentiate into a variety of cell types (39, 40), and may overcome the limitations of differentiated cells (36) when used for tissue engineering. Pluripotent by definition, stem cells can be derived from embryonic, fetal, or adult (or postnatal somatic) tissue (39). Stem cells are the current preferred cell source for tissue-engineering endeavors and regenerative medicine therapies due to their high potency and capacity for expansion (41). Contemporary research efforts have focused on adult stem cells or progenitor cells for tissue-engineering purposes. The use of embryonic and fetal tissue, although providing pluripotent stem cells with high proliferative potential, raises potential ethical issues as well as safety concerns over tumorigenic potential (42, 43). Adult derived stem cells, which are found among differentiated cells, have been isolated from an increasingly varied number of tissues over the past decade such as bone marrow (i.e., mesenchymal stem cells and hematopoietic stem cells), adipose [adipose-derived stem cells (44)], epithelial (epithelial-derived stem cells), and umbilical cord (cord blood stem cells) tissue (43). Developments made in isolation and culture of adult derived stem cells have improved cell yield during harvest. Subsequent research has focused on manipulating proliferation and differentiation into the desired cell type (45). Adult derived stem cells can only divide a finite number of times and accumulate genetic changes that can limit their supply for tissue-engineering purposes. The discovery of induced pluripotent stem cells (iPSCs) by Takahashi and Yamanaka (46) introduced the idea that a mature differentiated cell could be reverted to a state of pluripotency and multilineage potential (Figure 4). While this process, which has been shown to be possible with human cells, creates a potentially limitless source of easily accessible stem cells, it is not without drawbacks (47). Reprograming the cells has raised questions about epigenetic effects in particular, with a number of papers purporting to show DNA errors that have arisen during the process of inducing pluripotency (47, 48). Questions have also been raised regarding immunogenicity of engineered tissues with iPSCs implanted into a genetically identical mouse able to provoke an unexpected T cell-mediated immune response (49).

**Figure 5:**
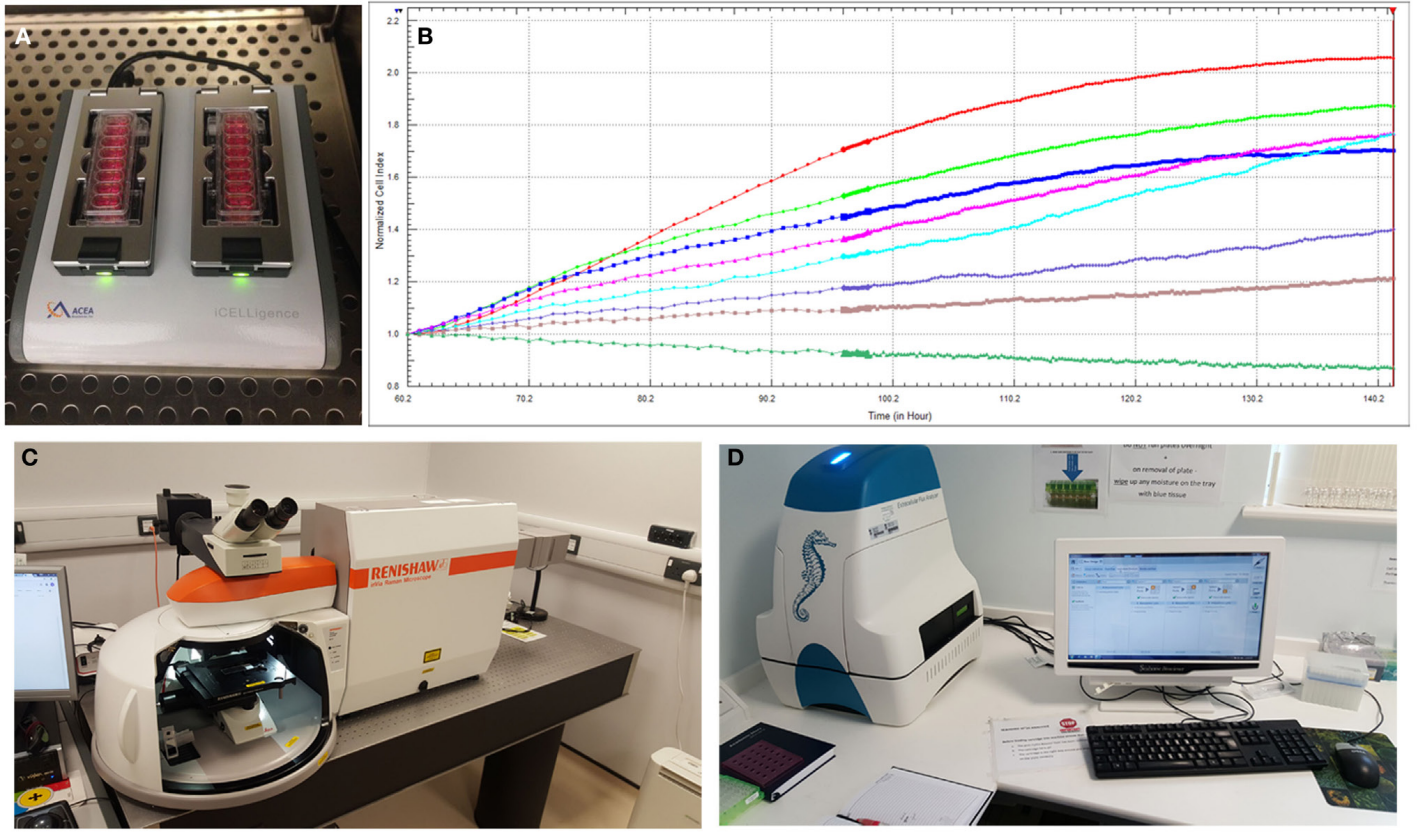
**Advanced technologies for monitoring cell behavior and survival**. **(A)** ICELLigence impedance based cell assay machine. **(B)** Proliferation curves at different cell seeding densities generated by iCELLigence. **(C)** The Renishaw inVia confocal Raman microscope allows identification of stem cells based on the scattering of photons due to vibrations of molecular bonds. **(D)** Seahorse XF^e^24 Extracellular Flux Analyzer is used for measurement of cellular bioenergetics.

To better understand the behavior of specific cell types and their utility for tissue-engineering purposes, there is an increased reliance on advanced technologies (41) to monitor cell phenotype, migration, proliferation, migration, and differentiation both *in vitro* and *in vivo*. Impedance-based systems such as iCELLigence system (ACEA Biosciences) as well as Seahorse XF^e^24 Extracellular Flux Analyzer are allowing real-time monitoring of cellular processes and offer distinct and important advantages over traditional endpoint assays (Figure 5). Contemporary imaging modalities such as two photon excited fluorescence microscopy (50) and Raman spectroscopy (51), both with high resolution and depth of penetration (>100 nm, 300 nm and 1 mm, 0.4 mm, respectively) are giving researchers clearer insights into the behavior of different cell types (Figure 5).

Further complications arise from the wide donor-to-donor variation in the behavior of cells, particularly stem cell populations, that has become apparent. Human adipose-derived stem cells, an increasingly prevalent source of adult stem cells for studies in tissue engineering, exhibit high donor-to-donor variability with regard to proliferation and differentiation characteristics, and this is not explained simply by donor age (52).

Currently, there is no consensus on the *ideal* cell source for tissue-engineering purposes. A thorough understanding of the advantages and disadvantages of each cell type is crucial to decide on cell selection and the optimal culture conditions in order to engineer specific tissue types.

##### Scaffold Choices

An appropriate scaffold is crucial to any tissue-engineering strategy. The ideal scaffold provides a framework for cell growth and development, allowing cells to attach, migrate, proliferate, and differentiate while facilitating cellular reorganization into a functional 3D network (Table 2).

**Table 2:** **Advantages and disadvantages of biomaterials utilized currently as scaffolds in tissue engineering**.

Scoffold class	Scoffold subtype	Macrostructure	Microstructure	Chemical composition	Advantages	Disadvantages
Synthetic	Polylactic acid (PLA)	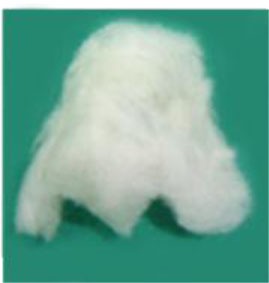	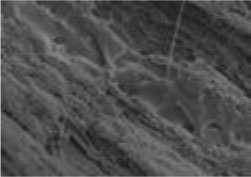	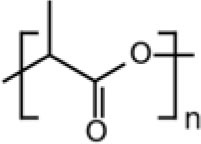	More predictable and reproducible mechanical and physical properties High tensile strength, degradation rate, and elastic modulus (7) More readily available Relatively inexpensive	Immune reaction Lack biological cues (8) Toxicity Infections
	Polyglycolic acid (PGA)	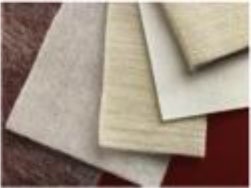	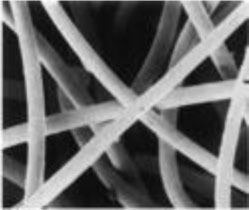	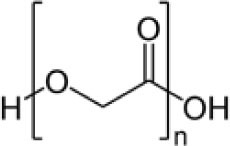		
	Polyethylene glycol derivatives (PEG)	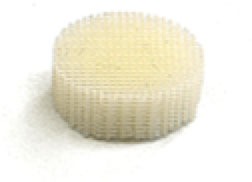	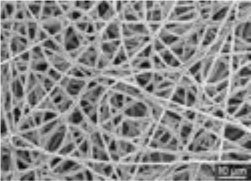	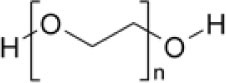		
Biological	Fibrin	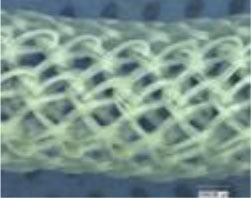	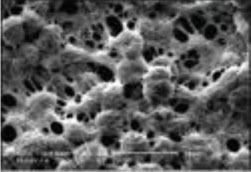	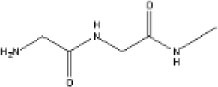	Biocompatibility Cell-controlled degradability Intrinsic cellular interaction Hydrated environment Non-toxic Mucoadhesive Cytocompatible (9)	Batch variations Limited range of mechanical properties Less reproducible Costly Specific processing conditions (10)
	Elastin	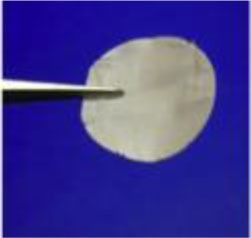	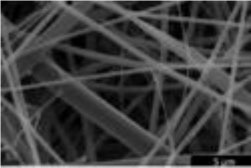	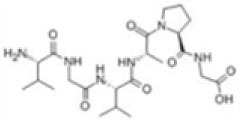		
	Collagen	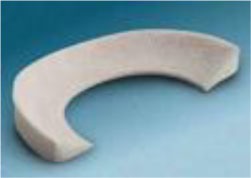	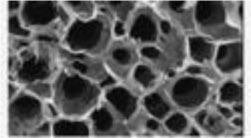	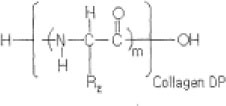		
	Alginate	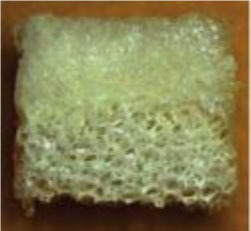	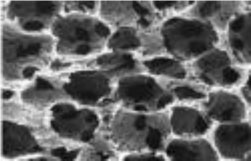	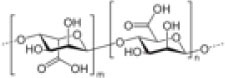		
	Agarose	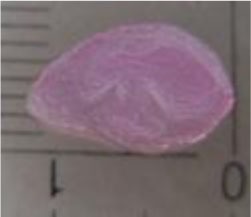	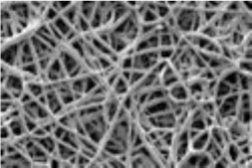	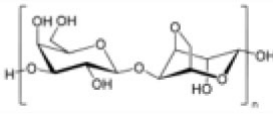		

Desirable characteristics of scaffolds include the following:

- Biomimetic (53)
- Biodegradable (with site specific absorption kinetics) (54–56)
- Appropriate mechanical strength
- Optimal micropores: enabling vascularization and allowing metabolic needs to be met (oxygen and waste product transfer)
- Biocompatible (57)
- Non-immunogenic (58)
- Versatile with regard to manufacturing methods
- Functionalization potential (59)
- 3D control of macroarchitecture
- Various nano- and micro-topographies, stiffnesses, and microenvironments appropriate to the proliferation, migration, and maturation of native or engrafted cells
- Suitable for clinical grade sterilization
- Suitable for industrial production.

Scaffolds are generally classified as biological (organic) or synthetic (inorganic). Engineering of the cell-scaffold construct can be undertaken *in vitro* in a bioreactor or *in vivo* by implanting the construct into the body. Advances in engineering, material science, and biomanufacturing technologies have enabled the design and development of more complex scaffolds using self assembly (60), computer modeling, bioprinting, and nanotechnology (60–62). “Functionalized,” “decorated,” or “smart” biomaterials that incorporate of biomolecular moieties on the surface, aim to orchestrate, and optimize the attachment and growth of cells and the synthesis of new tissue (61, 63). Scaffold size is largely limited by the lack of effective vascularization. Most successful work in the field focuses on understanding native tissue constituents and microarchitecture to allow accurate reproduction of functional tissue (64).

##### Environment

Consideration of the biophysiochemical 3D environment is crucial for tissue engineering. Cells not only require a scaffold for structural and biological support but also require an environment that provides the correct combination of growth supplements, differentiation signals, perfusion of nutrients, gaseous/waste exchange, pH regulation, and mechanical forces. The metabolic requirements of different tissues are varied and dictate the perfusion, gaseous/waste exchange, pH, and mechanical environment required. There is an increasing awareness that molecular and mechanical signaling is pivotal in the growth and differentiation of tissue-engineered constructs, and in addition to well-known growth factors such as bone morphogenetic proteins, vascular epithelial growth factor, basic fibroblast growth factor FGF-2, and transforming growth factor-β (65), “induction factors,” including oxygen tension (66–68), mechanical (69), and electrical stimulation (70), guide subsequent proliferation and differentiation of cells. Cells participate in a web of multidirectional interactions within their niches and tissues of residence [interacting with various nanotopographically sized cues (71)]. This has implications during biomimetic tissue engineering, where the cellular environment (biomolecules), scaffold topography, and other external factors (mechanical and electrical stimulation) require regulation. This is complicated by the fact that cells not only respond to multiple stimuli but also have a direct impact on the environment themselves.

**Figure 6:**
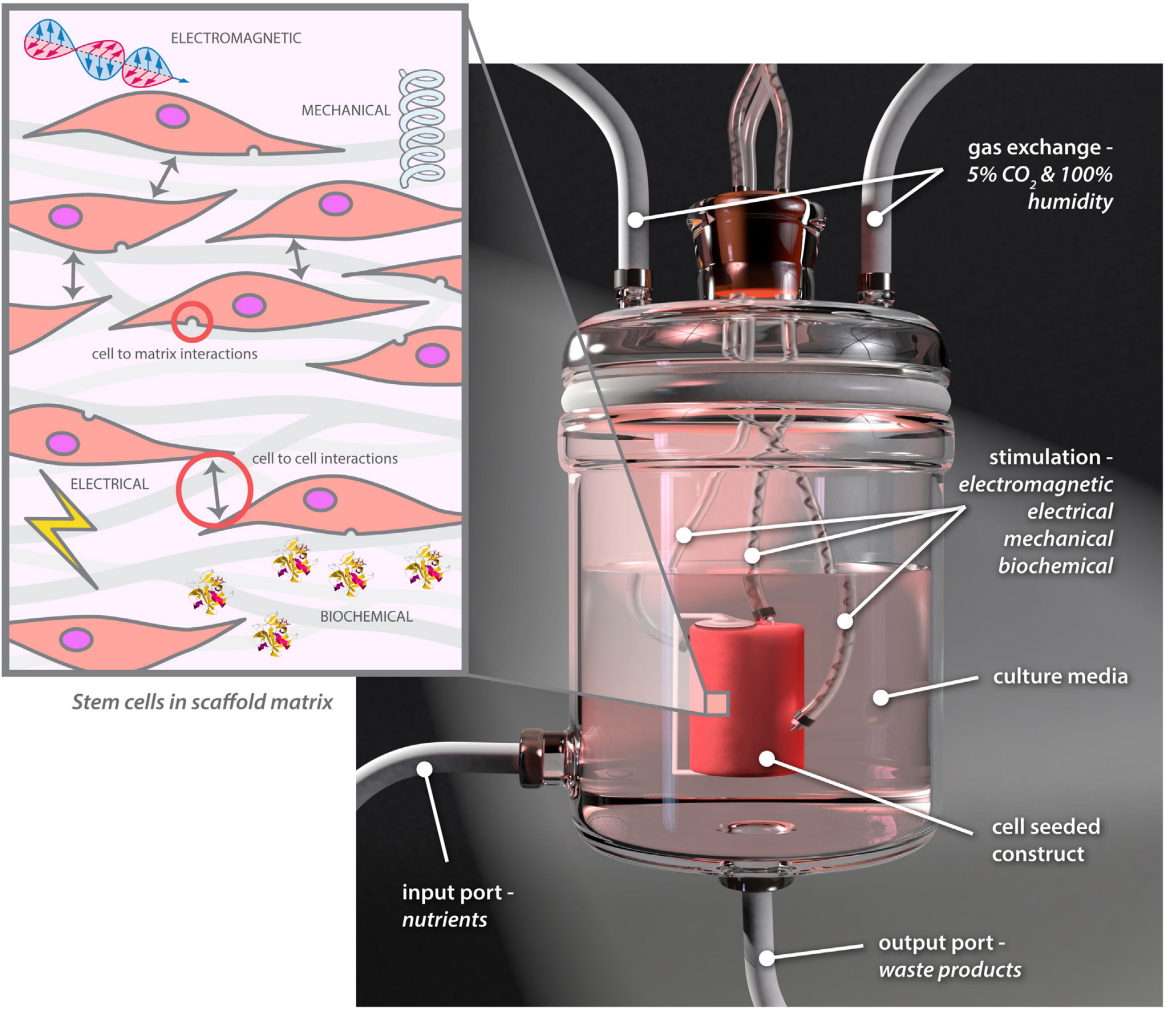
**Different environmental stimuli and the fundamental components of bioreactor technology**.

Research in this field is at the interface of cell biologists, engineers, materials scientists, and clinicians and is expanding rapidly. Multidisciplinary teams are working on bioreactor technology, which is vital for *in vitro* tissue engineering. Optimal conditions can to a great extent be applied and controlled through the use of bioreactors to mimic required conditions (72), and specialized bioreactors to help engineer a range of tissues have been developed in recent years (73, 74). Bioreactors are increasingly being used to provide more complex environments, exposing cells to a range of controllable electrical, electromagnetic, biomolecular, and mechanical cues while varying cell–cell or cell–matrix interactions (Figure 6) (73).

## Current Barriers to Translation

**Figure 7:**
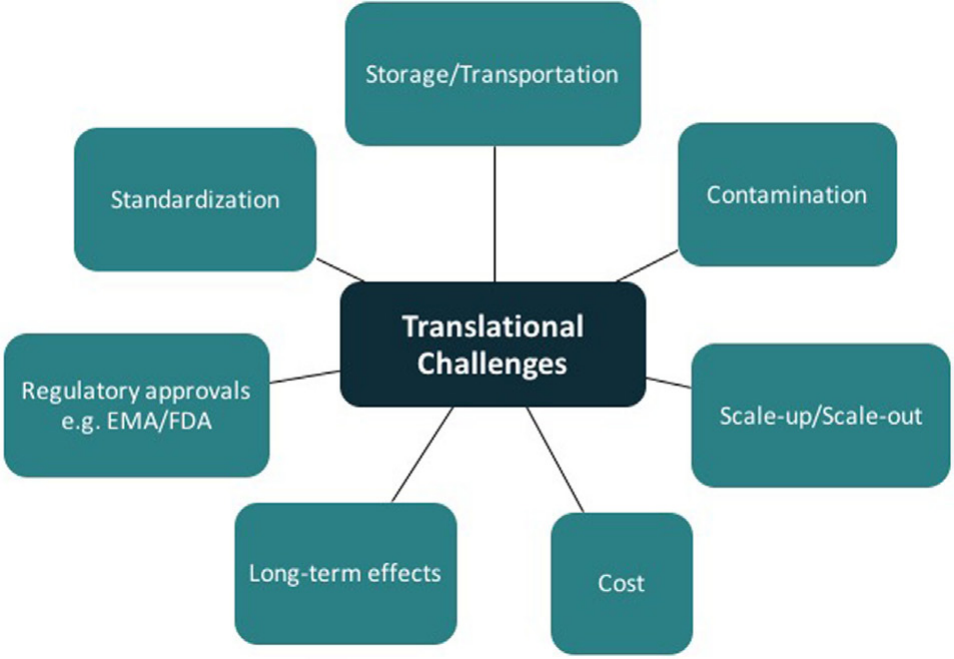
**Barriers to translation in tissue engineering**.

Tissue engineering has the potential for major clinical impact in plastic and reconstructive surgery. Some products using tissue-engineering concepts are already on the market; however, the panacea of functional vascularized composite tissue-engineered constructs is still theoretical. Translation of good basic science research from the laboratory to clinical reality remains a considerable challenge (Figure 7). In addition to meeting the scientific challenges of engineering durable and functional tissue for implantation into patients (75), one must also navigate the complex regulatory processes. The regulations controlling the delivery of stem cell therapeutics to the clinic parallel many of those developed for the pharmaceutical industry (76, 77). Guidelines governing the development of cell-based products can be found on websites for the U.S. Food and Drug Administration (FDA),^1^ the European Medicines Agency,^2^ and related governmental regulatory authorities. The United States Pharmacopeia is an internationally recognized resource defining the currently accepted industry standards for product purity, potency, and quality assurance.^3^ These targets are hard to meet outside a large, well-equipped commercial enterprise. Many research laboratories attached to clinical facilities do not produce mesenchymal stem cells in accordance with the criteria for either GLP or the more stringent cGMP, both requiring strict operational and certification records relating to all laboratory equipment and reagents used in the cell manufacture process (1, 4, 5).

A clear understanding of the manufacturing workflow is required to allow autologous and allogeneic tissue-engineered product integration into clinical practice (78). Once cells are obtained from a donor, they must be stored in specialized banks and both “scaled-up” (increasing batch size) and “scaled out” (increasing number of batches). There are several challenges in mass production alone including the monitoring of product yield and ensuring purity, potency, and viability throughout the process. Mass production of autologous tissue requires a facility allowing multiple, parallel, patient-specific production lines. Where scaffolds are required, testing and maintenance of quality attributes needs to be undertaken prior to seeding (78). Additional challenges include storage/transportation, contamination, and obtaining regulatory approval.

The long-term safety, efficacy, and functionality of the products also need to be closely assessed (79). Practical considerations that need to be contemplated include the storage environment and shelf life of the manufactured products.

It is also of interest that tissue-engineered products do not easily conform to either of the traditional Food and Drug Administration classification: biologics or devices (80, 81). Combined scaffold and cell-containing devices may be in more than one classification category. For devices, a single confirmatory study is often sufficient for FDA approval. If the product is regulated as a biologic, it must be reviewed and approved by the FDA Center for Biologics Evaluation and Research. If regulated as a drug, several further phases are required prior to FDA approval. Although these regulatory processes above present significant challenges, many countries do have streamlined regulatory processes that might reduce the obstacles faced (82).

## Tissue Engineering—Where are We Now?

Stable and physiologically relevant (74) tissue replacement with composite engineered tissue remains elusive. Allograft transplantation has been an exciting development for the reconstructive surgeon, but the requirement for long-term immunosuppressive therapy (83), health-care infrastructure, and funding streams means it is not mainstream practice (Table 3). Tissue engineering is a promising alternative and has yielded small successes so far. Atala et al. were the first to report tissue-engineered constructs being used in patients (84). This was followed by several reports between 2008 and 2014 in a range of tissues including the trachea (85, 86), urethra (87), and nasal cartilage (88). Results have been varied; tracheal work is currently under investigation due to the deaths of three out of six patients and for nasal cartilage in particular, there was a question mark whether the tissue was replaced by scar or native tissue (89). The current significant barriers to translation for large-volume tissue replacement are the inability to produce “physiologicallty relevant tissue” (74) and difficulties with vascularization (24). Small constructs may succeed based on local angiogenesis (84, 87); however, the metabolic needs of the implanted cells in larger constructs means prevascularisation or the use of vascular pedicles is likely to be necessary (87).

**Table 3:** **Successful applications of tissue-engineered constructs in humans**.

Organ/tissue	No. of patients	Cell source	Outcomes	Reference
Bladder	7	Bladder urothelial and muscle cells	Improved volume and compliance with no metabolic consequences at mean 46 months follow-up	Atala et al. (84)
Trachea	1	Recipient MSCs	Functional airway with a normal appearance and mechanical properties at 4 months	Macchiarini et al. (85)
Urethra	5	Muscle and epithelial cells	Maintenance of wide urethral calibers without strictures, normal architecture on biopsy at 3 months following implantation	Raya-Rivera et al. (87)
Nasal cartilage	5	Autologous nasal chondrocytes	Good structural stability and respiratory function after 1 year	Fulco et al. (88)
Vaginal organs	4	Vulval biopsy—epithelial and muscle cells	Tri-layered structure on biopsy with phenotypically normal smooth muscle and epithelia with follow-up up to 8 years	Raya-Rivera et al. (90)

## Conclusion and Future Perspectives

Surgical reconstruction using bioengineered tissues has the potential to revolutionize clinical practice. To be successful, one must be able to generate tissue constructs *in vitro* that are morphologically and functionally similar to native tissues. There has been a steady increase in basic science activity in cell therapy and a growing portfolio of cell therapy trials; however, this has not translated to commercial products available for clinical use. To achieve clinical translation, a multidisciplinary approach that successfully integrates engineering and biological methodologies is necessary. Ethical, regulatory, financial, and clinical considerations all present challenges in the translation of tissue-engineered constructs from the laboratory to mainstream clinical practice. Even though The Medical Research Council states that regenerative medicine and tissue engineering “holds the promise of revolutionizing patient care in the 21st century” (91) and that stem cell therapy is viewed as a future “game changer” by the plastic surgery community (92), many are yet to be convinced of this potential within the NHS, with major concerns involving cost-effectiveness, efficacy, reimbursement, and regulation (93). There is little doubt that tissue engineering offers great potential to reduce patient morbidity and mortality, and only co-ordinated and prolonged liaison between clinicians, scientists, and industry will move this from potential to reality.

## Glossary

### Cell Biology

**Differentiation**—The process by which a cell becomes specialized in order to perform a specific function.

**Commitment**—When a cell becomes dedicated to a specific lineage.

**Potency**—The array of commitment opportunities available to a cell.

**Totipotent**—Cells capable of differentiating into any body cell type in addition to extraembryonic or placental cells.

**Multipotent**—Cells capable of differentiating into multiple cell types along one lineage (e.g., hematopoietic stem cells).

**Pluripotent**—Cells that may differentiate into tissues derived from all three germ cell layers.

**Unipotent**—Cells only capable of differentiating into one cell type (e.g., spermatogonial stem cells).

**Clonal**—A population of identical cells derived from the same cell.

**Polyclonal**—A population of cells derived from multiple clones.

**Progenitor**—A cell that has limited potency, but is able to differentiate to another cell type, or differentiate to its target cell lineage.

**Embryonic stem cells**—Embryonically derived pluripotent cells that are obtained from the inner cell mass.

**Induced pluripotent stem cells**—Differentiated cells that are reverted to their pluripotent state via a set of transcription factors.

**Autologous**—Cells or tissues obtained from the same individual.

**Allogeneic**—Cells or tissues obtained from a different individual of the same species.

**Xenogeneic**—Cells or tissues obtained from a different species.

**Extracellular matrix**—Biomolecules synthesized by the cell to provide a suitable environment to support surrounding cells and maintain tissue integrity in response to biochemical and mechanical cues.

### Biomaterials/Scaffolds

**Scaffold**—A 3D biomaterial construct that defines the geometry of the replacement tissue and provides environmental cues that promote tissue regeneration.

**Biomimetic**—Human-made substances, e.g., scaffolds that imitate nature.

**Functionalization**—The modification of scaffolds with bioactive material to enhance the biocompatibility of the scaffold.

**Nanotechnology**—Technology that deals with dimensions and tolerances of less than 100 nm, especially the manipulation of individual atoms and molecules.

**Biomolecular factors**—Biomolecular factors include growth factors, transcription factors, and components of the extracellular matrix.

**Mechanical factors**—External environmental stimuli such as forces generated during everyday movement.

### Manufacturing

**Bioprinting**—the process of generating spatially controlled cell patterns using 3D printing technologies.

**Bioreactor**—System in which conditions are closely controlled to permit or induce certain behavior in living cells or tissues.

**Good Laboratory Practice (GLP)**—System of management controls for laboratories conducting research to ensure consistency, reliability, reproducibility, and high quality of chemical (including pharmaceutical) tests.

**Good Manufacturing Practice (GMP) guidelines**—Regulatory guidelines that outline specific requirements for the handling and processing of human tissue, ensuring safe products of reliable quality.

**Scale-out**—Increasing the number of batches of an engineered product.

**Scale-up**—Increasing batch size on an engineered product.

## Author Contributions

SA-H and ZJ completed a literature search and contributed to preparing the manuscript. AA-S, EC, and AI contributed ideas and content to the manuscript. SD, AH, CT, and CA critically revised the manuscript. IW conceived, contributed to the preparation of, and critically revised the manuscript. All the authors read and approved the final manuscript and agreed to be accountable for all aspects of the work.

## Conflict of Interest Statement

The authors declare that the research was conducted in the absence of any commercial or financial relationships that could be construed as a potential conflict of interest.

## Footnotes

^1^http://www.fda.gov/.

^2^http://www.ema.europa.eu/ema.

^3^http://www.usp.org/.
